# COVID-19 and people with intellectual disability: impacts of a pandemic

**DOI:** 10.1017/ipm.2020.45

**Published:** 2020-05-14

**Authors:** K. Courtenay, B. Perera

**Affiliations:** Barnet Enfield and Haringey Mental Health NHS Trust, London N15 3TH, UK

**Keywords:** Autism, COVID-19, intellectual disability, pandemic

## Abstract

The impacts of the COVID-19 pandemic affect all groups in society. People with intellectual disability (ID) are especially vulnerable to the physical, mental and social effects of the pandemic. Cognitive impairments can limit understanding of information to protect them relying on carers to be vigilant on their behalf during quarantine. Restrictions on usual activities are likely to induce mental stress especially among those who are autistic leading to an escalation in challenging behaviours, risk of placement breakdown and increased the use of psychotropic medication. People with ID are vulnerable to exploitation by others where the usual community supports no longer function to protect them. In future pandemics, it is important that lessons are learned from the impacts COVID-19 have on people with ID. Collecting the evidence through a rigorous approach should help to empower people with ID and their carers to face future outbreaks of infectious diseases.

## Introduction

The global COVID-19 pandemic has moved swiftly across the globe infecting millions and testing the health care systems of countries. In the course of the pandemic, the vulnerabilities of certain groups of people have been highlighted such as the elderly, pregnant women and the homeless (Kirby, 2020; Qiao, 2020; WHO, 2020). Less apparent has been the plight of people with intellectual disability (ID) who have a range of vulnerabilities that include health problems, mental disorders and social disadvantage (Emerson & Hatton, 2008). Meeting the need to protect people with ID from infection and to support those infected is a challenge to care services often because of the unique characteristics of people with ID to adapt to new circumstances. The concern of families and carers is that people with ID may be forgotten as the pandemic unfolds (Silverman, 2020) and that responses should not exclude people with disabilities (Berger *et al.* 2020).

## Risk of infection

People with ID are at greater risk of infection for a range of reasons that include physical health problems, social circumstances and limitations in understanding (Grier *et al.* 2020). The prevalence of comorbid physical disorders is higher among people with ID, and their life expectancy is lower than that of the general population with a standardised mortality ratio of 3.18 (Glover *et al.* 2017). People with ID and genetic disorders may suffer from hereditary cardiac, inborn errors of metabolisms or respiratory conditions. Respiratory infections are the leading cause of death in people with ID (O’Leary *et al.* 2018) especially among people with Down syndrome (O’Leary *et al.* 2018). The level of obesity is higher among people with ID raising their risk of experiencing severe forms of infection with COVID-19 (Biswas *et al.* 2010; Perera *et al.* 2020). Overall, the prevalence of physical and mental disorders in people with ID is higher than in the general population (Cooper *et al.* 2007; Perera *et al.* 2020).

People with ID live in a range of models of accommodation such as care homes, supported living placements or hospital in-patient services (Perera & Courtenay, 2018) that are essentially congregated settings. Many live with family members who are elderly parent carers whose health status is often compromised (Taggart *et al.* 2012). In community settings, people with ID often require high levels of support from family members or paid carers that increases their level of social contact. Gimenez *et al.* (2010) reported on the impact of an outbreak of H1N1 infection in an institution for people with ID that led to a high rate of mortality among the residents attributed to the low level of vaccination among them. The carers posed the greatest risk of infection to the residents as vectors of the infection. The findings were supported by Yen *et al.* (2012) who detected a low rate of uptake of seasonal influenza of 23% compared with 38% of the elderly in the general population in Taiwan.

In general, people with ID live in the community with support from family members or paid carers depending on their level of support need. Those with mild ID (IQ 50–70) are likely to require less support to undertake activities of daily living compared with those with moderate to severe levels of ID. Their participation in community activities is good with access to community activities and some people are engaged in paid employment. People with ID often follow their own routines and need to be prepared for changes. If not, sudden changes can increase their level of anxiety causing behavioural challenges and potentially mental health conditions.

In times of pandemic, people with ID are likely to have difficulty in advocating for themselves and rely on others to keep them safe from infection. For those with mild ID who function in the community with little support, their cognitive ability may hinder their adherence to public health measures to reduce the spread, for example, self-isolation, handwashing or physical distancing from others. Such social behavioural demands can be difficult to understand and to implement especially for those with behavioural challenges such as spitting that can pose increased risk to other people with ID and their carers. In pursuing such measures, it may be difficult for carers to support people for whom curtailing their limited freedoms could be problematic especially where there has been a disruption to their personal routines, for example, attending regular day-time activities. For people with autism, self-isolating and physical distancing can heighten their level of stress because of a change in habits. They may become over-focused and subsequently overwhelmed by the amount of information related to COVID-19 in the media and on social media. Such behaviour may heighten their levels of anxiety and paranoid thinking leading to difficulties in their behaviour thus further reducing the ability to practise social distancing behaviours.

## Access to information

Relevant and informative information on infection is crucial in supporting people with ID to adhere to behaviours to reduce the spread of infection. They may rely on others to inform them using information in accessible formats to help them understand the infection and how to reduce the risks of contracting it. Providing accessible information to carers and people with ID is essential especially where literacy skills are limited (MENCAP, 2020).

With the trend in moving from hospital settings to living in community settings, people with mild ID are living more independently in the community. Achieving more independent living with low levels of support is positive under stable circumstances but with a stark and rapid change in societal expectations as in a pandemic, people’s ability to adapt and be flexible is tested potentially stressing their personal resources to adjust to new patterns of social behaviour expected by all. Quarantine is likely to be difficult for people with ID to tolerate and compounded by not understanding its importance and the implications of not adhering to restrictions. It is important therefore to use effective communication methods to inform people that are likely to have positive effects on their resilience in enduring the constraints on their usual activities (Brookes *et al.* 2020).

## Responses to the pandemic for people with ID

In a short space of time, non-governmental organisations have responded to support people and carers by producing materials on COVID-19 and for people with ID. Specific guidance from family carers (The CBF, 2020), voluntary groups (MENCAP, 2020) and guidance for professionals (LD Senate, 2020) have been disseminated and in accessible formats to facilitate ease of understanding. Voluntary organisations advocate on behalf of people with ID who cannot speak for themselves and have been effective in raising awareness among Government agents of the plight of people with ID by challenging assumptions made about them. The NICE Guideline, NG159, initially suggested using the Clinical Frailty Scale to assess a person’s suitability for escalation to more invasive medical treatments. The Guideline was challenged by carers who protested that young people with ID and COVID-19 could suffer because of biased clinical application of the scale denying them medical care on the basis of their disability (BBC News, 2020; NICE, 2020).

## Impact on others

The impact on families and carers is especially heightened where the usual supports of residential schools, day services or respite care have been withdrawn due to the pandemic. Support from local authorities and government agencies is especially needed to support families at a time when they are likely to experience great strain in providing 24-h care that was usually shared with paid carers. The impact will be on the finances of families and their well-being and mental health. Support organisations recognise the need for extra support for families without which the risk of breakdown is high potentially leading to the need for hospital admission because of increasing challenging behaviour (MENCAP, 2020; The CBF, 2020).

## Mental health and ID

In addition to stress associated with the fear of contracting the illness, social distancing and quarantine measures in place can have an impact on the mental health of some people (Pfefferbaum & North, 2020). The mental health of people with ID can be affected in similar ways, if possibly with greater impact because of the demands of quarantine potentially triggering problem behaviours. Autism or ADHD in a person with ID may worsen the situation where their usual routines cannot be fulfilled and with restrictions on their physical environment (Narzisi, 2020). Carers of people with ID may need to self-isolate that can lead to breakdown of the person’s care network resulting in exacerbations of behavioural problems.

Increased anxiety and paranoia can be seen in exceptional circumstances such as in the current lockdown. People with ID and autism can become obsessed about information related to COVID-19 which would be understandable given that obsessional thinking and obsessive compulsive disorders are common among people with autism (Meier *et al.* 2015). It may lead to excessive levels of anxiety and paranoid thinking resulting in behavioural challenges. Comorbid obsessive compulsive disorders could be compounded by the need for scrupulous personal hygiene. These triggers can mount to high levels of overwhelming stress leading to mental illness. Many behavioural and psychological interventions cannot be implemented due to significantly reduced face to face work by health and social care staff further complicating access to appropriate interventions.

## Supporting people infected with COVID-19

The rate of infection in communities in many countries is not certain in the absence of strategic and proactive testing for the virus generally. The evidence of harm to elderly people in community residential homes highlights the rapid impact infection can have on groups of people living together (Tan & Seetharaman, 2020). There are little data on the rates of infection among people with ID in the community or in in-patient services. Where infection is present, support staff need to adjust their practices, but many feel let down by their health care system to adequately protect them by providing personal protective equipment (PPE) and the skills to care for infected people (Iacobucci, 2020). Care staff are expected to acquire new skills normally practised by nurses and to implement them with the aim of supporting people who are ill to remain in the community to ease pressure on in-patient services. Practices such as barrier nursing and infection control measures are required to manage people with infection and to limit the spread of the virus. Applying these measures can be a challenge when caring for people with ID who are young and healthy and who may not understand the importance of adhering to infection control. Services may be challenged too where staff are absent due to illness or the need to self-isolate. Access to personal protective equipment, as recommended by the WHO (2020), for carers in the community has become an issue because of global shortages of equipment (Park *et al.* 2020).

## Specialist clinical services

The specialist clinical services for people with ID need to adapt to the changing environment in which they deliver care whether through community services or specialist in-patient services. Clinicians engaging in physical distancing are advised not to meet with people to reduce the risk of infection and have to rely on new methods of providing care. The use of technology is beneficial in helping clinician and patient to visualise each other (Greenhalgh *et al.* 2020) but not everyone with ID has access to technology and elderly carers may not be skilled in using it thus creating a barrier to their family members receiving direct care.

The issue of problem behaviour as a result of lockdown measures in the pandemic may be difficult for carers to manage using the person’s current positive behavioural support plan and plans should be revised during the pandemic. This may lead to a greater reliance on medication to support a person to remain in their current residence that is contrary to initiatives to reduce the use of psychotropic medication among people with ID (Branford *et al.* 2019). In-patient services are affected by restrictions on access to community supports that are often essential elements of rehabilitating people for community living.

## Mental health legislation

As a consequence of the pandemic and its impact on clinical practice, the usual legal processes for detaining people have had to adapt in order to reduce the risk of transmission. General advice on keeping staff safe from infection is available, and the UK Government has passed legislation (only enacted in Northern Ireland to date) to enable detentions and Mental Health Review Tribunals to continue to function during the pandemic (DHSC, 2020). The processes for reviewing detentions are undertaken using technology to enable Tribunals to confer with legal representatives and clinicians (NHS England, 2020). Such measures enable the implementation of legislation under extraordinary circumstances, but detained people are entitled to review of their detentions to ensure that measures in place do not contravene their human rights. It will be important for when the pandemic has passed that the interim novel processes return to standard practice. Where future enhancements to legal processes are suggested as a result of the experience under the pandemic, they should not be at the cost of a detained person’s rights.

## Advance care planning

Preparing to care for people whose risk of death is high is a challenge to carers and family members. Advance Care Planning assists carers to help families and the person with ID to prepare for such an eventuality. It can require difficult conversations with relatively young people on their perceptions and wishes on end-of-life care. Treatment escalation plans are an essential part of advance planning in helping carers to recognise when the need for greater medical care is required and to consider end-of-life care (Ravi *et al.* 2020).

## Safeguarding

The risk of harm from others through various forms of abuse is high among people with ID as evidenced by major scandals in Winterbourne View Hospital (Flynn, 2012) and Whorlton Hall (Murphy, 2019). Statutory structures are in place to protect people who are abused or at risk of abuse. The rise in domestic violence in the general population during the pandemic should alert services to the potential of greater risk to people with ID (WHO, 2020). Changes in work practices to comply with physical distancing and the use of remote communication may result in fewer opportunities for clinicians to meet with people to gain a sense of a person’s safety (Galea *et al.* 2020). The usual methods of assessing safety may not be feasible for clinicians and others in a person’s social network to undertake and other means of detection of abuse need to be developed to protect people from harm. Conducting safeguarding investigations that rely on collecting information on allegations of abuse may be affected during the pandemic and complicate the investigatory processes even further. There is likely to be greater reliance on using technology to conduct safeguarding interviews and where face to face contact is necessary, social distancing and using PPE will be essential.

## Future pandemics and people with ID

The immediate effects of the pandemic on people with ID are unknown at present since data on infection with COVID-19 specific to people with ID are not available. The long-term effects of the virus on the health of people with ID can only be speculative such as the potential impacts of COVID-19 on neonates and unborn children are uncertain. Will there be consequences on foetal development similar to the Zika virus epidemic where microcephaly with neurological impairments were reported (de Oliveira Melo *et al.* 2016)? Neonates testing positive for COVID-19 have recovered (Zeng *et al.* 2020), but the potential long-term developmental impacts of the virus on foetal development are unknown. Using evidence from other populations infected by other viruses is valid, but findings do not always apply to distinct populations. Monitoring people with ID infected by the virus through prospective collection of data is essential in order to grow the evidence base on how it affects them.

The social impacts of the pandemic are evident at present but what of the long-term consequences of it on health and well-being? The potential impact on population mental health is evident, but it is not certain how the experience of the pandemic will affect people with ID who may experience the social upheaval in unique ways to other populations. For clinical staff supporting people with ID, new ways of working are likely to be adopted optimising the use of technology in delivering clinical care that could lead to more efficient and streamlined services. Staff will require training in using new technology and new practices to ensure they are effective but also secure from breaches of confidentiality.

How will people with ID adjust to new practices with potentially fewer face to face meetings and more reliance on technological communication? It will be necessary for paid carers and family carers to have a workable level of IT literacy when interacting not just with health services but care services too. Technology will need to ensure that it is accessible to people with cognitive impairments and limited communication using software applications. Research in to the utility of adaptive technology will be required in order to learn what is effective and what people with ID prefer to use and how. Their participation in research in technology will be essential that should help to empower them in the long term.

The use of PPE could deter people from engaging in clinical care. Such changes are likely to test the resilience of people to adapt and may require extra support to adjust to changes in practice. It is likely, however, that using PPE will be necessary in future pandemics. Training people with ID on how it is used by their carers will help prepare for the social adaptations that need to happen when pandemics arise.

What lessons can be learned from the pandemic that will offer some protection against future pandemics? To offer the best protection in future pandemics, a good knowledge base on effective protective and treatment interventions of infectious agents is key. Such knowledge can only be developed through thorough and methodologically robust research approaches to expand the evidence on COVID-19 in people with ID. Protecting vulnerable people especially those with ID and their carers should be a priority in any planning for future pandemics. In-depth reviews of the spectrum of actions in response to the pandemic are required to learn from the global experience and to prepare and plan for the future. Educating carers and people with ID on the signs of infection with COVID-19 along with behavioural measures to reduce the spread of infection are important. Clinical ID services will play an important role in disseminating knowledge and educating on COVID-19. In order to learn about the impact of the pandemic in the lives of people with ID by capturing important and relevant experiences, a co-productive collaborative approach with families, carers and people with ID will be essential.

## Conclusion

COVID-19 has had profound effects on populations worldwide. Attention has focused on those most physically affected by the pandemic. The experiences of the pandemic by people with ID need to be elicited in order to understand the impacts on their lives and how they have protected themselves from infection. It is essential that we learn from the pandemic on how to protect people with ID on account of their inherent vulnerability to infection and to the social consequences of the measures put in place to manage the pandemic. When the time comes to review the course of the pandemic, it is important that people with ID and their carers are not ignored in order to ensure that they are empowered to face such occurrences in the future.
